# In Situ Chemical Modification of Thermoplastic Starch with Poly(L-lactide) and Poly(butylene succinate) for an Effectively Miscible Ternary Blend

**DOI:** 10.3390/polym14040825

**Published:** 2022-02-21

**Authors:** Piyawanee Jariyasakoolroj, Suwabun Chirachanchai

**Affiliations:** 1 Department of Packaging and Materials Technology, Faculty of Agro-Industry, Kasetsart University, Bangkok 10900, Thailand; 2 Center for Advanced Studies for Agriculture and Food (CASAF), KU Institute for Advanced Studies, Kasetsart University, Bangkok 10900, Thailand; 3 The Petroleum and Petrochemical College, Chulalongkorn University, Bangkok 10330, Thailand; suwabun.c@chula.ac.th; 4 Center of Excellence on Petrochemical and Materials Technology, Chulalongkorn University, Bangkok 10330, Thailand

**Keywords:** in situ ring-opening polymerization, poly(lactide), poly(butylene succinate), thermoplastic starch, ternary blend

## Abstract

Thermoplastic starch (TPS) is in situ ring-opening polymerized with L-lactide (L-LA) and directly condensed with a poly(butylene succinate) (PBS) prepolymer in an extruder using two different production pathways to demonstrate the concept “like dissolves like” in a miscible poly(lactide)/TPS/PBS (PLA/TPS/PBS) ternary blend. The TPS crystalline pattern changes from a V_H_-type to an E_H_-type after TPS modification with a hydrophobic-PLLA segment. Heteronuclear multiple-bond correlation confirmed the successful formation of PLLA-TPS-PBS copolymers via two different in situ chemical modification pathways (i.e., (I) step-by-step modification and (II) one-pot reaction). All obtained PLLA-TPS-PBS copolymers functioned as the miscible phase, enhancing PLA/PLLA-TPS-PBS/PBS ternary blend miscibility, especially the random structural PLLA-TPS-PBS-II copolymers created in an in situ one-pot reaction. However, the PLLA-TPS-PBS-I copolymers can enhance PBS crystallization only. While the random PLLA-TPS-PBS-II copolymers exhibit a homogeneous multi-phase dispersion and crystallization acceleration in both the PLA and PBS chains. Moreover, the storage modulus level of the PLA/PLLA-TPS-PBS-II/PBS ternary blend remains high with a downward temperature shift in the glass transition region, indicating a stronger and more flexible system. The practical achievement of in situ modified TPS and, consequently, a miscible PLA/PLLA-TPS-PBS/PBS ternary blend with favorable physical properties, reveal its potential application in both compostable and food contact packaging.

## 1. Introduction

In recent years, environmental concerns have prompted important changes in governmental and institutional policies. Biodegradable plastics have been widely employed to reduce plastic waste pollution, especially in disposable packaging [1]. PLA, PBS, and other biodegradable resins made through reliable, industrial-scale production methods are currently used to meet these environmental policies. PLA is derived from renewable resources (e.g., corn, beet, and cassava) that has attractive properties such as clarity, high tensile strength, and approval as food contact materials. PLA, however, is considered very brittle with low crystallization and low degradation rates [2,3]. Meanwhile, PBS is home-compostable, thermally stable, highly flexible with low glass transition temperature (T_g_), and has a high degree of crystallinity (40–60%). Unfortunately, PBS is expensive and opaque with poor melting strength and a limited processing window [4,5]. 

Another promising biodegradable plastic is thermoplastic starch (TPS), produced from plasticized starch using several possible plasticizers (e.g., water, glycerol, formamide, alcohols, and fatty acids) [6,7,8]. In the past decade, TPS has received much attention due to its natural abundance, toughness, and low cost. However, TPS is sensitive to moisture, leading to structural reorientation (i.e., retrogradation) during storage. This intrinsic characteristic reduces shelf-life, causing undesirable physical properties and surface stickiness. 

Melt blending is a simple approach with practical industrial processes that combines the unique and attractive characteristics of various polymers into a single material to overcome the limitations of each component [9]. PLA/TPS-blend research has focused on its renewability, compostability, and affordability [10]. However, water absorption and long-term storage issues limit the usage of PLA/TPS blends in current packaging applications [11,12,13]. To counter these issues, several studies have investigated combining PLA/TPS-based ternary blends with other hydrophobic biodegradable polymers, such as poly(ε-caprolactone) (PCL) [14,15,16,17,18,19], PBS [18,20], and poly(butylene adipate-co-terephthalate) (PBAT) [21,22,23]. 

Until now, PLA/TPS/PCL ternary blends have been researched extensively for their high ductility and thermal stability improvement due to PCL composition. Despite the fact that PCL is relatively expensive, it still has excellent biocompatibility potential to use in medical application. Recently, Koh et al. reported on interfacial tension considerations, regarding multicomponent compatibility in PLA- and TPS-based blends using PCL or PBS as a transitioning phase. Interfacial tension values between PLA and PBS phases, and PBS and TPS phases were the lowest, with reduced agglomeration for each component in the system, as compared to those of PCL/PLA and PCL/TPS blends. Additionally, the determined polarity of PBS (0.29) was between those of PLA (0.17) and TPS (0.53). Therefore, the interphase interfacial interactions of PLA, PBS, and TPS blends increased spontaneously [18]. 

As mentioned previously, TPS is relatively hydrophilic, while the biodegradable polyesters are highly hydrophobic; therefore, PLA/TPS-based ternary blends are immiscible and thermodynamically unstable. In the past, many reactive compatibilizers and PLA- and TPS-grafted copolymers were used to improve miscibility of the ternary blends. For instance, Zhen et al. used one-step reactive extrusion to produce a PLA/TPS/PBS ternary blend with maleic anhydride (MA) as the reactive compatibilizer. The miscibility of this PLA/TPS/PBS ternary blend only occurred in the amorphous region due to reduced water absorption, but phase separation still occurred at over 20 wt % PBS [20]. Carmona et al. found that methylene diphenyl diisocyanate (MDI) was a highly effective compatibilizer in PLA/TPS/PCL ternary blends, whereas MA without an organic peroxide initiator obstructed the compatibilization function. MDI urethane linkage between TPS and polyesters through one-step extrusion increased the melt strength and ductility of the ternary blend [24]. Nevertheless, the toxicity of diisocyanate compatibilizer became an issue. Moreover, the synthesized graft copolymers also improved the miscibility in PLA/TPS-based blends in other research [14,25,26]. For example, Wootthikanokkhan et al. prepared PLA grafted with MA modified TPS (PLA-g-MTPS) using a two-step reaction (i.e., MA was in situ reacted with TPS, and subsequently grafted onto PLA chain with the presence of peroxide as an initiator). PLA-g-MTPS replaced MA as a compatibilizer, and significantly improved the miscibility and toughness in the binary blend [26]. 

Unfortunately, using initiators or inorganic catalysts or the complicated copolymer synthesis was concerned for material contact safety and incorporation into industrial-scale production. Moreover, most graft copolymer research focused on combining two different kinds of polymer chains. However, the miscibility improvement and detailed structural analyses of biodegradable ternary blends using a tri-component graft copolymer as a ‘miscibilized’ phase has rarely been investigated. This prompted our deep analysis of triblock copolymers functioning as a ‘miscibilized’ phase for PLA/TPS/PBS ternary blends to create fully compostable, practical, and cost-effective materials that meets food-safe packaging requirements for upscale production.

In this study, TPS was in situ chemically modified with L-lactide (L-LA) and PBS prepolymer at different feeding molar ratios to obtain PLLA-TPS-PBS copolymers. These were further mixed with a PLA/PBS 50/50 blend to investigate interfacial adhesion improvement among PLA, TPS, and PBS phases. Two different routes of in situ TPS modification were used to compare triblock copolymer formation effectiveness and multiphase miscibility improvement. The detailed structure of the in situ chemically modified TPS was analyzed by two-dimensional nuclear magnetic resonance spectroscopy (2D-NMR) and gel permeation chromatography (GPC). Furthermore, the crystallization behaviors and dynamic mechanical analysis of PLA/PLLA-TPS-PBS/PBS ternary blends were investigated.

## 2. Materials and Methods

### 2.1. Materials

Commercial PLA (Ingeo™ Biopolymer 2003D) and PBS (BioPBS™-FZ91PM) resins were purchased from NatureWorks LLC, Minnetonka, MN, USA, and PTT MCC Biochem Co., Ltd., Rayong, Thailand, respectively. L-LA, cassava starch, and glycerol were purchased from Chiang Mai University, ETC International Trading Co., Ltd., and Asian Scientific Co., Ltd., Bangkok, Thailand, respectively. Sodium hydroxide (NaOH) was purchased from Sigma-Aldrich Corp., Saint Louis, MI, USA. Acetic acid 96% (AR grade), chloroform (HPLC-grade), and hydrochloric acid 37% (AR grade) were obtained from RCI Labscan Ltd. Deuterated dimethyl sulfoxide-d6 and deuterated chloroform were purchased from Merck Millipore Corp., Darmstadt, Germany. All chemicals were used without any further purification.

### 2.2. Preparation of PLLA-TPS-PBS Copolymers

Cassava starch was plasticized with glycerol at a weight ratio of 70/30 with a Labtech Engineering LTE 20–40 twin-screw extruder (Labtech Engineering, Samut Prakan, Thailand). Pure TPS was produced at a temperature ranging from 100–150 °C (between the feeder to die zones) with a screw speed of 40–70 rpm. 

Meanwhile, the PBS resin with a number average (M_n_) and a weight average (M_w_) molecular weight of ~80 kDa and ~175 kDa, respectively, (PBS1) was soaked in 1 molar NaOH at 30 °C for 24 h before naturalization with 20 mL of a 5% acetic acid solution and distilled water to prepare PBS2 (M_n_ ~40 kDa and M_w_ ~85 kDa). All PBS resins with different molecular weights were dried in a hot air oven at 50 °C for 24 h.

To begin, a PLLA-TPS-PBS copolymer was prepared through pathway I with step-by-step in situ ring-opening polymerization and direct condensation (PLLA-TPS-PBS-I). The obtained pure TPS was further modified with L-LA to form a PLLA-TPS copolymer in the twin-screw extruder at 100–120 °C with a screw speed of 50 rpm. The crude PLLA-TPS copolymer was purified with distilled water several times to remove unreacted L-LA. Next, the obtained PLLA-TPS copolymer was directly in situ condensed with two different molecular weight (MW) PBS resins at a 50/50 weight ratio in the twin screw extruder at 150–160 °C with a screw speed of 40–50 rpm. 

Another PLLA-TPS-PBS copolymer was produced by pathway II with an in situ one-pot reaction. The obtained pure TPS was in situ copolymerized simultaneously with L-LA and PBS using the twin-screw extruder to form PLLA-TPS-PBS-II. All obtained PLLA-TPS-PBS copolymers were washed with distilled water to remove unreacted L-LA and dried at 50 °C for 24 h. L-LA/starch/PBS feeding molar ratios were varied as indicated in Table 1.

### 2.3. Preparation of PLA/PLLA-TPS-PBS/PBS Ternary Blend

The commercial PLA and PBS resins were blended with PLLA-TPS-PBS-I and PLLA-TPS-PBS-II copolymers using the twin-screw extruder in the temperature range of 150–170 °C. The studied PLA/PLLA-TPS-PBS/PBS weight ratio was fixed at 35/30/35. After that, the PLA/PLLA-TPS-PBS/PBS blend was blown into a film using a Labtech Engineering LE-25–30/C single-screw extruder equipped with a Labtech Engineering LF-400 blown film unit (Labtech Engineering, Samut Prakan, Thailand).

### 2.4. Characterization

#### 2.4.1. Structural Analyses

The in situ ring-opening polymerization of PLLA-TPS copolymer formation and the in situ condensation reaction of the PLLA-TPS-PBS copolymers were confirmed by a Bruker Biospin Advance 500 nuclear magnetic resonance (NMR) spectrometer (Bruker, Billerica, MA, USA). Deuterated dimethyl sulfoxide-*d_6_* (DMSO-*d_6_*) and deuterated chloroform (CDCl_3_) were used as solvents for the PLLA-TPS and PLLA-TPS-PBS copolymers, respectively. 

**Scheme 1:**
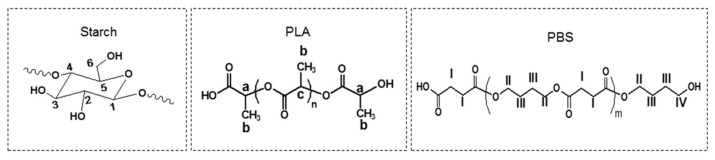
Chemical structures of pure starch, PLA and PBS.

^1^H and ^13^C NMR (500 MHz, DMSO-*d_6_*, δ) for starch (Scheme 1): 3.30–3.85 (br, CH-2-CH-5), 3.78 (s, CH_2_-6), and 5.1 (s, CH-1) assigned to protons of anhydroglucose unit (AGU); 60–65 (C-6), 65–75 (C-2-C-5), and 100 (C-1) assigned to carbon-atom in AGU. 

^1^H and ^13^C NMR (500 MHz, CDCl_3_, δ) for PLA (Scheme 1): 1.52 (s, CH_3_-b), 4.38 (br, CH-a) and 5.21 (s, CH-c) assigned to protons of lactic acid unit; 18–19 (CH_3_-b), 68.7 (CH-c), and 170 (C=O) assigned to carbon-atom in lactic acid unit. 

^1^H and ^13^C NMR (500 MHz, CDCl_3_, δ) for PBS (Scheme 1): 2.64 (s, CH_2_-I) in succinic acid unit, and 30 (CH_2_-CH_2_-I) and 172 (C=O) assigned to carbon-atom in succinic acid unit; 1.78 (s, CH_2_-III) and 4.14 (br, CH_2_-II) assigned to protons, and 26 (-CH_2_-CH_2_-III) and 68 (O-CH_2_-II) assigned to carbon-atom in butanediol unit.

The M_n_, M_w_, and polydispersity index (PDI) of PBS were determined by a Shimadzu Prominence liquid chromatography equipped with the LC-20AD and CTO-20A system, and Shodex GPC K-802.5, 803, 804, and 805 columns (Kyoto, Japan). Polystyrene standards and chloroform as the eluent with a flow rate of 1 mL/min were used. Moreover, PLLA-TPS and PLLA-TPS-PBS crystalline pattern changes were analyzed with a Bruker D8 Advance X-ray diffractometer (Bruker, Billerica, MA, USA) using a Cu-K_α_ radiation source. 

#### 2.4.2. Contact Angle Measurement and Morphological Observation 

PLLA-TPS’ surface hydrophobicity was determined with an LMS Instruments FA17 water contact angle machine (Midview City, Singapore). The final measured water contact angle was recorded after 15 s and each sample was measured 5 times. 

PLA/PLLA-TPS-PBS/PBS blend cross-sectional morphologies were evaluated with a JEOL JSM-IT300 scanning electron microscope (SEM) (Tokyo, Japan). The sample surface was sputter-coated with gold for 3 min.

#### 2.4.3. Thermal Properties 

A Mettler Toledo DSC1 STARe differential scanning calorimeter was employed (Greifensee, Switzerland). All samples were sealed in a 40 μL aluminium pan before heating from −50 °C to 200 °C (heat-cool-heat conditions) at a rate of 5 °C/min under a nitrogen flow of 50 mL/min. Furthermore, the X_c_ values of PLA and PBS were calculated using Equations (1) and (2), respectively.

X_c,PLA_ = [(ΔH_m,PLA_ − ΔH_cc,PLA_)/(93.1 J/g × W_PLA_)] × 100% (1)

X_c,PBS_ = [(ΔH_m,PBS_)/(110.3 J/g × W_PBS_)] × 100% (2)

where ΔH_m,PLA_ and ΔH_cc,PLA_ represent enthalpy changes of PLA fusion and PLA cold crystallization, respectively. ΔH_m,PBS_ is the enthalpy of crystalline PBS. W_PLA_ and W_PBS_ are the PLA and PBS weight fractions in the PLA/PLLA-TPS-PBS/PBS ternary blend, and 93.1 J/g and 110.3 J/g are the enthalpy changes of 100% crystalline PLA and PBS, respectively, as reported by Fischer et al. [27] and Gigli et al. [28].

#### 2.4.4. Dynamic Mechanical Analysis 

Mechanical properties of the obtained PLA/PLLA-TPS-PBS/PBS films were studied using a Netzsch GABO EPLEXOR^®^ (Selb, Germany) dynamic mechanical thermal analyzer in tension mode at a frequency of 1 Hz and 0.1% strain. The samples were heated from 0 °C to 140 °C with a heating rate of 5 °C/min.

## 3. Results and Discussion

### 3.1. Structural Analyses of the PLLA-TPS and PLLA-TPS-PBS Copolymers

**Scheme 2:**
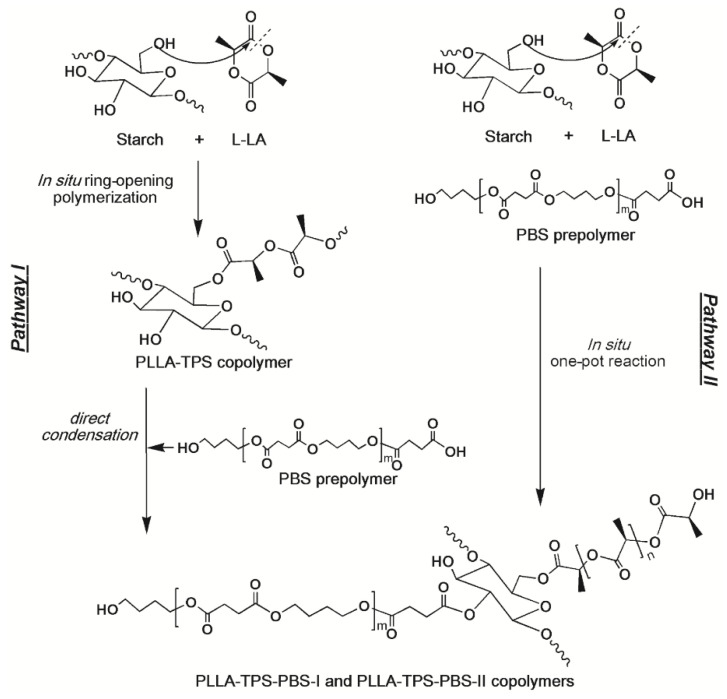
PLLA-TPS-PBS copolymers prepared by pathway I and II.

**Figure 1:**
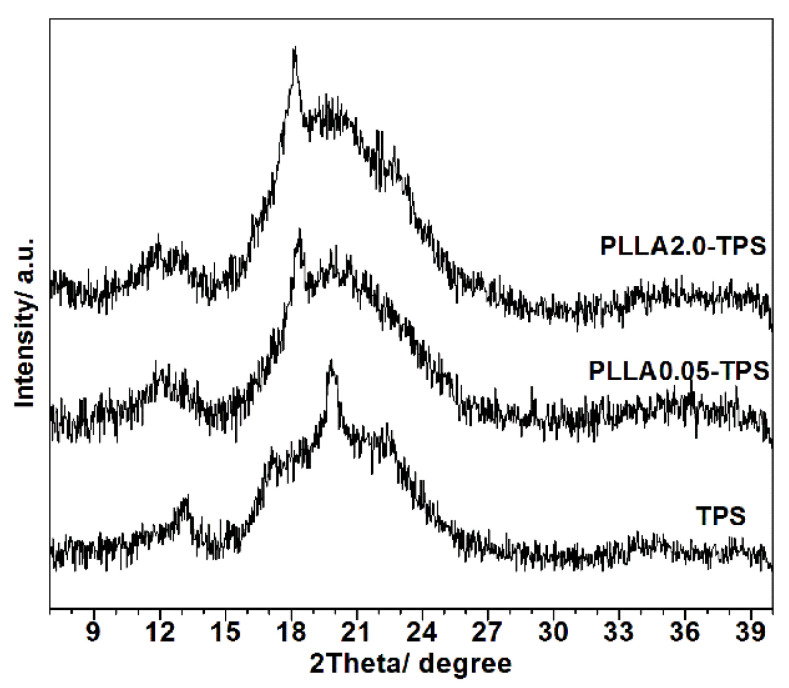
WAXD patterns of pure TPS and PLLA-TPS copolymers by pathway I.

Cassava starch was first processed to form TPS. The TPS was then modified with L-LA through in situ ring-opening polymerization in the twin-screw extruder. A reactive hydroxyl group on the carbon-6 (CH_2_-6) AGU unit was anticipated to attack the carbonyl group of L-LA (and, consequently, the ring-opening of L-LA) to form the PLLA-TPS copolymer in pathway I, as proposed in Scheme 2. The in situ ring-opening polymerization was preliminarily traced by wide-angle X-ray diffraction (WAXD) patterns (Figure 1) and water contact angle measurements for confirmation of success.

As shown in Figure 1, pure TPS diffraction peaks are 13.3°, 19.9°, and 22.4° 2θ, referred to processing-induced crystalline or a V_H_-type crystalline pattern. These pure TPS diffraction patterns were also observed and reported by Yeh et al. [7] and Teixeira et al. [29]. After L-LA was in situ ring-opening polymerized with pure TPS in pathway I, diffraction peaks were observed at 12.2°, 13.0° and 18.4° 2θ. This indicated a so-called E_H_-type pattern where highly crystalline starch granules undergo a high degree of disruption by reacting with L-LA in the PLLA segment, as observed by Biliaderis [30]. Similarly, Chen et al. [31] synthesized a starch-grafted PLLA copolymer through L-LA ring-opening graft polymerization, and found a broad lactic acid diffraction peak at 18.9° 2θ. The shifting of diffraction peaks in Figure 1 confirmed the success of PLLA-TPS formation through pathway I. Moreover, the peak broadened and shifted to 18.2° 2θ when the feeding molar ratio was increased to 0.20/1 L-LA/starch. This implied that increasing the PLLA chain length obstructed the arrangement of the amylose chain. 

**Figure 2:**
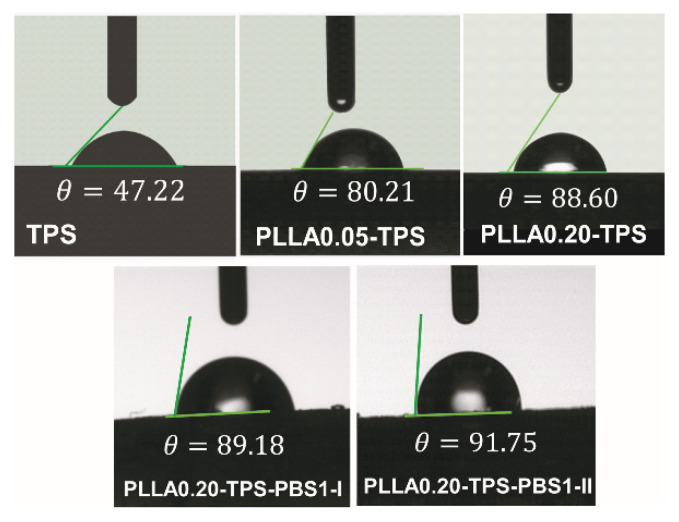
Water contact angle of the PLLA-TPS and PLLA-TPS-PBS copolymers.

Hydrophobicity improvement of in situ copolymerized TPS was determined by measuring the water contact angle. When TPS was in situ ring-opening polymerized with L-LA at L-LA/starch feeding molar ratios of 0.05/1 and 0.20/1, the water contact angle on the TPS-based surface was enhanced from 47.22° to 80.21° and 88.60°, respectively. It could be summarized that the greater the L-LA content, the more hydrophobic PLLA chains became in PLLA-TPS molecules obtained from pathway I. Furthermore, the PLLA-TPS-PBS-I and PLLA-TPS-PBS-II average water contact angles gained from step-by-step modification (pathway I) and one-pot reaction (pathway II), respectively, were similar, reaching approximately 90°, as shown in Figure 2.

**Figure 3:**
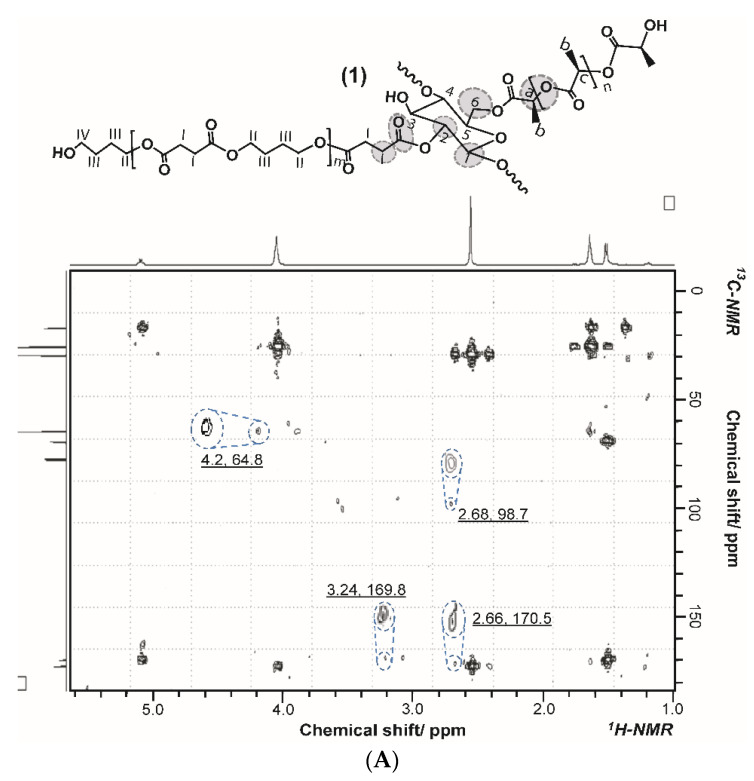

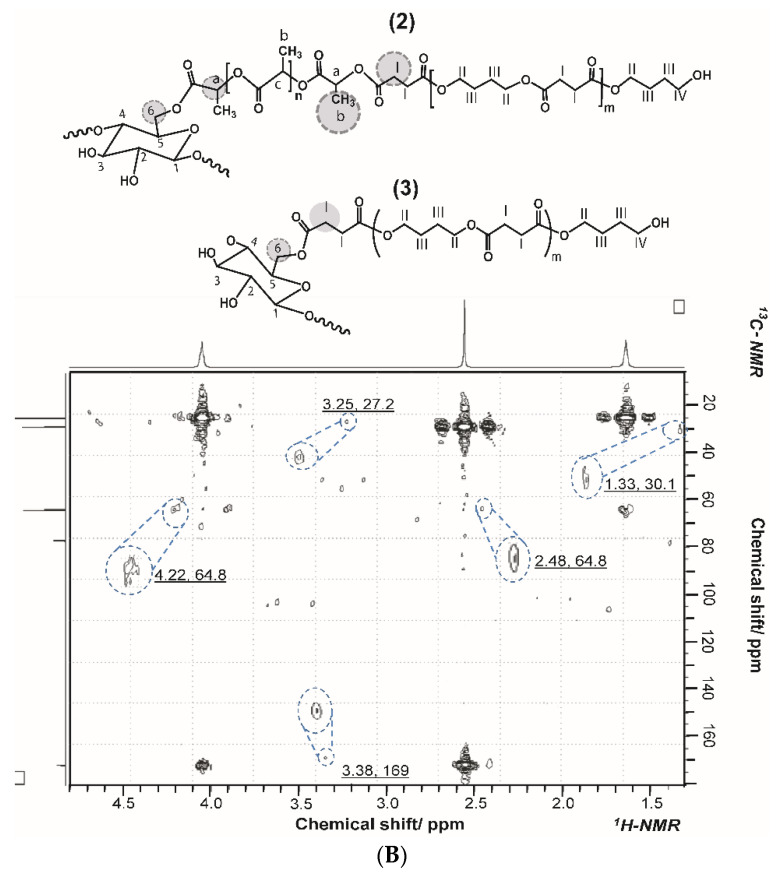
(**A**) ^1^H-^13^C HMBC correlation spectra of PLLA0.2-TPS-PBS2–I copolymer in CDCl_3_ solvent. (**B**) ^1^H-^13^C HMBC correlation spectra of PLLA0.2-TPS-PBS2–II copolymer in CDCl_3_ solvent.

Furthermore, the correlation of protons’ chemical shifts with carbons separated by two or more chemical bonds was utilized to trace the covalent bond formation via in situ ring-opening polymerization and direct condensation (i.e., pathways I and II, respectively) using a 2D HMBC (Heteronuclear Multiple Bond Correlation) NMR experiment. However, the partially dissolved PLLA-TPS-PBS copolymer in the CDCl_3_ solvent limited this experiment due to presence of the highly hydrophobic PLLA and PBS with the less hydrophobic TPS moieties. The correlation between the methine (CH-a) proton on the PLLA chain at 4.2 ppm and the CH_2_-6 carbon of the AGU unit at 64.8 ppm was observed. The protons of the PBS methylene (CH_2_-I) at 2.68 ppm and AGU methine (CH-2) at 3.24 ppm correlated with carbons of the PBS carbonyl group (C=O) at 170.5 ppm and 169.8 ppm, respectively. These crosspeaks confirm that the PLLA-TPS-PBS-I structure formed as proposed (**1**) in Figure 3A. The ^1^H and ^13^C NMR spectra of PLLA-TPS-PBS copolymers were shown in Figure S1.

For PLLA-TPS-PBS-II (Figure 3B), the successful formation of proposed structure (1) in the in situ one-pot reaction was confirmed by the chemical shift correlation between (i) the PLLA methine proton (CH-a) at 4.22 ppm and the AGU carbon-6 (CH_2_-6) at 64.8 ppm, and (ii) the AGU methine proton of carbon-2 (CH-2) at 3.38 ppm and the PBS carbonyl carbon (C=O) at 169 ppm. Additionally, the PLLA methyl protons (CH_3_-b) and PBS methylene carbon (CH_2_-I) correlation at 1.33 ppm and at 30.1 ppm, respectively, implied condensation between the PLLA chain and the PBS prepolymer. Therefore, the PLLA-TPS-PBS-II system was contaminated by the copolymer with the proposed structure (2). Furthermore, the cross peaks of the PBS methylene protons (CH_2_-I) at 2.48 ppm and the AGU carbon-6 (CH_2_-6) at 64.8 ppm indicated that the PLLA-TPS-PBS-II structure could randomly form as the proposed structure (3). The differences between the in situ chemical modification steps played a key role in the different structures of PLLA-TPS-PBS copolymers obtained. TPS that had been first modified with L-LA by pathway I avoided either the transesterification or condensation between the PLLA and PBS chains themselves, as observed in TPS modification by pathway II. Similarly, a random sequence distribution was also found throughout the graft copolymer of polyesters produced by one-step or in situ ring-opening polymerization and esterification, as reported by Zeng et al. [32] and Choi et al. [33]. 

### 3.2. Cross-Sectional Morphologies

#### 3.2.1. PLLA-TPS-PBS Copolymers

**Figure 4:**
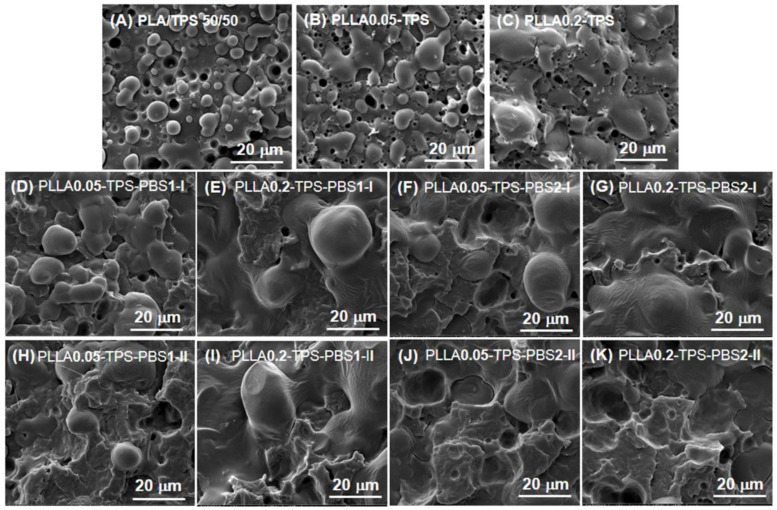
SEM micrographs of (**A**) PLA/TPS blend, (**B**,**C**) PLLA-TPS copolymers by pathway I, and PLLA-TPS-PBS copolymers by pathways I (**D**–**G**) and II (**H**–**K**).

Due to the differences in hydrophobicity and crystallization rate of TPS, PLA, and PBS phases, the phase separation in simple PLA/TPS-based blends always occurs with the presence of starch granules. After TPS was in situ ring-opening L-LA, the starch granules were destructed, and the continuous interfacial phases were expanded, according to an increased L-LA/starch feeding molar ratio (Figure 4B,C). 

In this study, the miscibility improvement from PLLA-TPS-PBS copolymers in PLA/PBS blends was proven by SEM. Figure 4D–G,H–K shows PLLA-TPS-PBS copolymer morphologies produced through pathways I and II, respectively. The starch granules are still obviously presented in both PLLA-TPS-PBS-I and PLLA-TPS-PBS-II copolymers. However, the TPS phase fusion in the polymeric matrices and the destruction of starch granular structure can be observed and increased with higher L-LA feeding molar ratios from 0.05/1 to 0.20/1 L-LA/starch. Moreover, it was noted that the phase continuity of modified TPS according to pathway I was clearly obtained; in particular PLLA0.2-TPS-PBS2-I (Figure 4G) produced from a high L-LA feeding molar ratio of 0.20, combined with short PBS chains. In contrast, the phase continuity of PLLA-TPS-PBS-II improved independently from the L-LA feeding molar ratio and the PBS chain length. The expansion of starch granule size after grafting and the encapsulation of starch granules with ring-opening polymerized PLLA and PCL chains were also revealed by Canché–Escamilla et al. [34], Rutot et al. [35], and Cuevas–Carballo et al. [36]. 

Nevertheless, less agglomerated starch granules were found on the rough PLLA-TPS-PBS-II surface. This can be explained by random PLLA-TPS-PBS-II structures obtained from the in situ one-pot reaction. These structures obstruct the inter- and intramolecular hydrogen bonds in starch molecules, leading to broken starch granules and the enhancement of starch hydrophobicity. However, the more ordered structure of the PLLA-TPS-PBS-I obtained from step-by-step modification did not completely destroy starch granules. This implies that the highly hydrophobic PLLA and PBS chains only modified on the starch surface. Therefore, the sequence of in situ TPS modification steps by pathways I and II, and the PBS chain length influenced the morphologies of PLLA-TPS-PBS copolymers. This could play a key role in the miscibility enhancement of the PLA/TPS/PBS ternary blend.

#### 3.2.2. PLA/PLLA-TPS-PBS/PBS Ternary Blends

**Figure 5:**
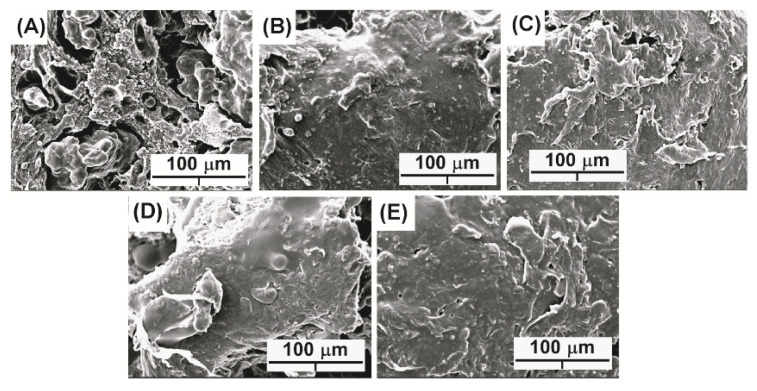
SEM micrographs of (**A**) PLA/TPS/PBS; (**B**) PLA/PLLA0.05-TPS-PBS1–I/PBS; (**C**) PLA/PLLA0.05-TPS-PBS2–I/PBS; (**D**) PLA/PLLA0.2-TPS-PBS1–I/PBS; and (**E**) PLA/PLLA0.2-TPS-PBS2–I/PBS ternary blends.

After that, the obtained PLLA-TPS-PBS-I and PLLA-TPS-PBS-II copolymers were further blended with commercial PLA and PBS resins at a PLA/PLLA-TPS-PBS/PBS blending weight ratio of 35/30/35. When applying PLLA-TPS-PBS-I to replace pure TPS in the PLA/PBS blend (Figure 5B–E), the homogeneous surfaces clearly improved as compared to those of the PLA/TPS/PBS blend (Figure 5A). The reduced interfacial tension among the PLA, PBS, and TPS phases was achieved by replacing TPS with the PLLA-TPS-PBS-I copolymer as an interphase tuning molecule, resulting in a miscible ternary blend. Identically, several PLA-g-starch and PLA-g-TPS graft copolymers effectively improved the miscibility in both binary and ternary as investigated by Wootthikanokkhan et al. [26], Zeng et al. [32] and Noivoil and Yoksan [37]. 

Fewer gaps were found in the PLA and PBS matrices; however, the surfaces of the ternary blends with PLLA-TPS-PBS-I were still relatively rough. There was TPS phase agglomeration in the ternary blends, consisting of PLLA-TPS-PBS-I copolymer with the long PBS chain (i.e., PLLA0.05-TPS-PBS1 (Figure 5B) and PLLA0.2-TPS-PBS1 (Figure 5D)), as compared to those of the PLLA-TPS-PBS-I copolymer with the short PBS chain. This suggests an incompatibility between PLA and PBS phases since the long PBS1 chain grafted on the PLLA-TPS-PBS-I molecule had poor interfacial adherence with the PLA-rich phase in the ternary blend. From a previous study, the PLA/PBS blend with the higher 20 wt % PBS content was thermodynamically incompatible, and consequently, the discrete minor phase formed an “island-like” domain embedded in a major phase matrix, as precisely discussed by Wu et al. [38].

**Figure 6:**
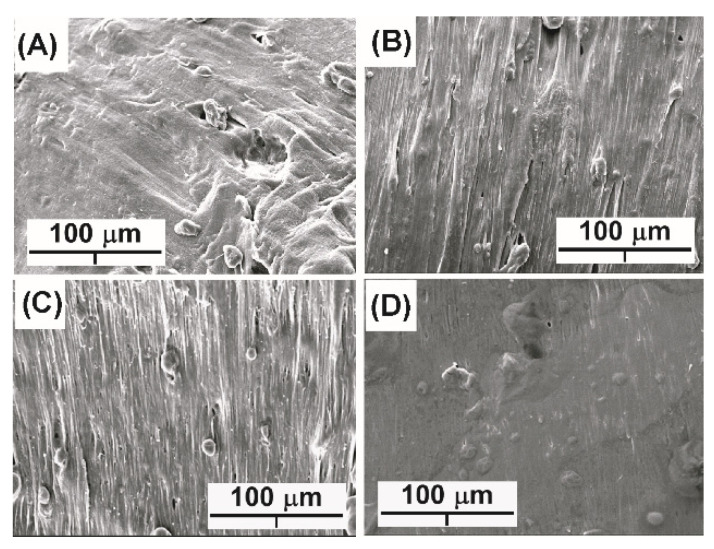
SEM micrographs of (**A**) PLA/PLLA0.05-TPS-PBS1–II/PBS; (**B**) PLA/PLLA0.05-TPS-PBS2–II/PBS; (**C**) PLA/PLLA0.2-TPS-PBS1–II/PBS; and (**D**) PLA/PLLA0.2-TPS-PBS2–II/PBS ternary blends.

In the case of PLLA-TPS-PBS-II, the miscibility also improved for the PLA/PLLA-TPS-PBS-II/PBS blend. The starch granule size decreased considerably and became well-dispersed. The surface smoothness was higher for the PLA/PLLA-TPS-PBS-II/PBS blend compared to the PLA/PLLA-TPS-PBS-I/PBS blend, especially in the PLA/PLLA0.2-TPS-PBS2–II/PBS blend (Figure 6D). Notably, this suggested that the obtained random PLLA-TPS-PBS-II structures (2 and 3) promoted TPS, PLA, and PBS multiphase miscibility. Additionally, the TPS copolymerized with a high L-LA feeding molar ratio and the short PBS chain length were optimal for enhancing the homogeneity of the PLA/PLLA-TPS-PBS/PBS ternary blend at a PLA/PBS weight ratio of 50/50.

### 3.3. Thermal Properties of the PLA/PLLA-TPS-PBS/PBS Blends

**Figure 7:**
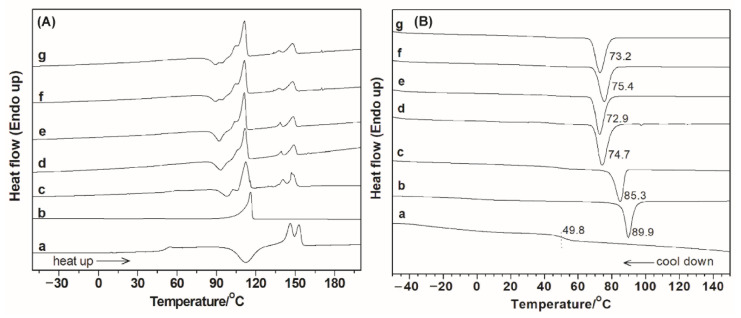
DSC thermograms of (**A**) second heat and (**B**) first cooling scans of (a) PLA, (b) PBS, (c) PLA/TPS/PBS, (d) PLA/PLLA0.05-TPS-PBS1–I/PBS, (e) PLA/PLLA0.05-TPS-PBS2–I/PBS, (f) PLA/PLLA0.2-TPS-PBS1–I/PBS, and (g) PLA/PLLA0.2-TPS-PBS2–I/PBS.

Furthermore, the thermal properties of the obtained PLA/PLLA-TPS-PBS-I/PBS and PLA/PLLA-TPS-PBS-II/PBS 35/30/35 blends were evaluated by the DSC technique and are summarized in Table 2. In general, PLA has a low crystallization rate and is an almost completely amorphous material, having an X_c,PLA_ value of 3–4% with T_g_ at 53.9 °C and cold crystallization temperature (T_cc_) at 124.5 °C, respectively. Figure 7A shows two distinct melting points of PLA, denoting the presence of both imperfect (δ-form) and complete (α-form) crystalline phases.

When PLA was blended with 50 wt % PBS, the T_cc_ of PLA decreased to 104.5 °C with a X_c,PLA_ of 25.03%, whereas the melting and crystallization temperatures of PBS (T_m,PBS_ and T_c,PBS_, respectively) shifted slightly downward to 114.5 °C and to 85.3 °C, respectively, with a X_c,PBS_ of 35.72%. This indicated that the highly crystalline PBS induced PLA crystallization, as also found by Wang et al. [39] and Deng et al. [40]. For the PLA/TPS/PBS 35/30/35 ternary blend, the T_g_ and T_cc_ values of PLA were observed at 53.9 °C and at 98.6 °C, respectively, while X_c,PLA_ insignificantly increased (24.66%) compared to that of the PLA/PBS binary blend. This was caused by phase separation, as confirmed by the SEM micrograph (Figure 5A). Contrarily, Zhen et al. [20] reported a decreased T_g_ of PLA at ~45 °C in the PLA/TPS/PBS ternary blend due to the miscibility of the amorphous region. 

The crystallization behaviors of PLA and PBS in the PLA/PLLA-TPS-PBS/PBS ternary blends were directly impacted by (i) PLLA-TPS-PBS copolymers replacing pure TPS in the ternary blend; (ii) the copolymer random structure; (iii) the L-LA/starch feeding molar ratio; and (iv) the PBS chain length. For the PLLA-TPS-PBS-I copolymer with the 0.05 L-LA feeding molar ratio in the blend, the T_g_ and T_cc_ of PLA were approximately at 50 °C and at 93 °C, respectively, with separate T_m_s of PLA at ~140 °C and ~150 °C. Notably, the X_c,PLA_ dropped considerably to 17–20% while the X_c,PBS_ increased substantially to 60% and slightly shifted T_m_ of PBS to 111 °C. Additionally, the T_c,PBS_ of the PLA/PLLA0.05-TPS-PBS-I/PBS ternary blends obviously decreased to 73–75 °C, as shown in Figure 7B. This suggested that the PLLA-TPS-PBS grafted copolymer acted as an interphase tuning molecule to enhance interfacial interaction between the PLA and PBS phases. TPS in situ ring-opening polymerized with a low L-LA feeding molar ratio was produced by pathway I and interfered with the order-arrangement of PLA chains in the crystalline phase. These results can be explained by a thermodynamic hypothesis whereby the partially miscible amorphous TPS in binary and ternary blends decreased the chemical potential in ‘crystallizability’ of low crystallization-rate polymer (i.e., PLA), as shown in a previous study in PLA, PCL, and TPS blends [15]. Furthermore, a small shoulder assigned to PBS segment on PLLA-TPS-PBS-I copolymer is obvious at 104 °C. Supthanyakul et al. [41] also revealed a similar splitting of PBS melting peaks after applying a PLLA-b-PBS-b-PLLA triblock copolymer as a multi-functional additive in PLA/PBS blend. 

Remarkably, PLLA-TPS-PBS-I copolymers which were produced by a high L-LA feeding molar ratio of 0.20 broadened the T_cc_ of PLA with a bimodal distribution and shifted the first T_m_ of PLA to 138–140 °C. This could be due to contamination of the PLLA prepolymer with a certain chain length, which cannot be removed by simple purification. Moreover, the obtained X_c,PLA_ and X_c,PBS_ values in the PLA/PLLA-TPS-PBS-I/PBS blends remained in the range of 16–23% and 57–63%, respectively. The results pointed out that homogeneity improvement of the ternary blend by pathway I preferentially promoted PBS crystallization rather than that of the PLA phase. The unchanged X_c,PLA_ implied an increased interaction between the PLA and amorphous TPS phases, and subsequently limited the orderly alignment of PLA chains. PLA chain mobility for crystallization was also similarly limited in the PLA/TPS blend incorporated with oligo(lactic acid)-g-starch due to improved intermolecular interactions between PLA and TPS domain phases, as reported by Noivoil and Yoksan [37].

Notably, when PLA/PBS was blended with the random PLLA-TPS-PBS-II copolymers, the X_c,PLA_ and X_c,PBS_ were as high as 23–30% and 53–63%, respectively, with decreased T_cc,PLA_ (~90 °C). This implied that the substantially improved miscibility of the PLA/PLLA-TPS-PBS-II/PBS blend accelerated crystallization for both the PLA and PBS phases. This phenomenon agrees with the results of using random PBS-co-PLLA copolymer prepared by simple polycondensation of L-LA and esterification as a compatibilizer in the PLA/PBS blend. This contributed to the homogeneous multiphase, and consequently accelerated PLA and PBS crystallization [42]. Moreover, the fluctuation of the decreased T_c,PBS_ values was also found in the temperature range of 67.0–84.4 °C. It was caused by the random PLLA-TPS-PBS-II copolymer structures as the interphase tuning molecule in the ternary blend. The DSC thermograms of the PLA/PLLA-TPS-PBS-II/PBS blend are presented in Figure S2.

### 3.4. Dynamic Mechanical Analysis of of the PLA/PLLA-TPS-PBS/PBS Blends

**Figure 8:**
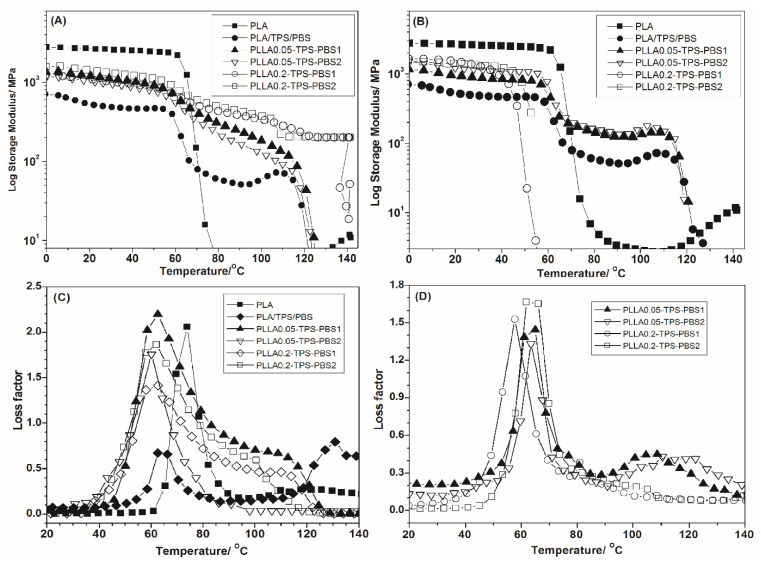
Storage modulus and loss factor (tan δ) as a function of temperature of PLA/PLLA-TPS-PBS/PBS films: (**A**,**C**) PLLA-TPS-PBS prepared by pathway I and (**B**,**D**) pathway II.

Furthermore, the DMA technique was used to characterize the mechanical properties of PLA/PLLA-TPS-PBS/PBS films as a function of temperature. Storage modulus (E′) and loss factor (tan δ) of PLA and the ternary blend films are shown in Figure 8. In general, PLA film is brittle; thus, the initial E’ below T_g_ is very high (2.75 GPa). At temperatures above the T_g_ value of PLA (>70 °C), the E’ dropped to zero rapidly and no longer regenerated crystals, as indicated by the inability to measure the E′ value. This phenomenon can be attributed to the low crystallization rate of PLA. The tan δ peak referred to the energy dissipation of PLA presents at 75 °C which correlated to T_g_ value determined by DSC thermogram. 

In the case of the PLA/TPS/PBS film, the initial E’ below the T_g_ of PLA decreased drastically to 0.75 GPa due to immiscibility in the ternary blend. TPS and PBS can, however, reduce the T_g_ of PLA and assist with PLA recrystallization. This was observed in the E’ transition region at approximately 60 °C with a slightly increased E’ above T_g_ due to severe phase separation in PLA/TPS/PBS ternary blend which caused material weakness. In addition, the tan *δ* peak shifts downward to 65 °C with a significantly decreased area under the tan *δ* peak, reflecting the low impact resistance of the immiscible ternary blend. 

Figure 8A shows the E′ of PLA/PLLA-TPS-PBS-I/PBS films, where the initial E’ values ranged from 1.0 to 1.7 GPa. These increased initial E’ values indicated the successful miscibility improvement, as compared to the PLA/TPS/PBS film. The observed tan *δ* peaks of the PLA/PLLA-TPS-PBS-I/PBS films shift to ~60 °C with increased tan *δ* peak height. This suggested the enhanced miscible multiphases resulted in improvement in the toughness of the PLA/PLLA-TPS-PBS/PBS ternary blend. 

Moreover, adding PLLA0.2-TPS-PBS2-I in the ternary blend also provided the highest initial E’, which correlated with the enhanced morphological phase continuity of TPS modified with high L-LA feeding molar ratio and short PBS segment. These can be summarized that the key factors in achieving improved strength of the PLA/PLLA-TPS-PBS/PBS ternary blend were (i) the presence of PLLA with bimodal chain lengths and (ii) the chain length balance of the PLLA and PBS segments in the in situ step-by-step modified TPS molecules. Additionally, the E’ above the T_g_ value of the PLA/PLLA-TPS-PBS-I/PBS films remained as high as 0.25–0.70 GPa, compared with those of pure PLA and PLA/TPS/PBS blend films (almost 0 GPa). It is worth mentioning that the TPS chemically bonded to the PLLA and PBS segments. This enhanced intermolecular interaction in the amorphous region of the ternary blend system and assisted mobilization of the amorphous PLA chain for thermal recrystallization. 

In the case of the PLA/PLLA-TPS-PBS-II/PBS films, the initial E’ shows a similar trend to that found in the PLA/PLLA-TPS-PBS-I/PBS films, but the random structures of the PLLA-TPS-PBS-II significantly decreased the thermal sensitivity of the glass transition region. This also implied the PLLA-TPS-PBS-II copolymer with a random structure improved flexibility which correlated to the downward shifting of tan δ peak in the range of 57–62 °C. In particular, adding PLLA0.2-TPS-PBS-II, with a high L-LA feeding molar ratio, clearly softened the ternary blends when below 60 °C, caused by the combining of the contaminated PLLA prepolymer with the random copolymer formation. Moreover, the E’ above the T_g_ value of the PLA/PLLA-TPS-PBS-II/PBS film was slightly lower than that with the PLLA-TPS-PBS-I addition, indicating the decreased intermolecular interaction between the PLA and PBS phases, due to blending with the random PLLA-TPS-PBS-II copolymers. The random structures of the in situ one-pot modified TPS through pathway II induced effective miscibility, and subsequently the completion of PLA crystallization with the retained strength of the PLA/TPS/PBS ternary blend. This result corresponded to the high X_c_ of PLA, the retained high initial E’, and slightly increased storage modulus. These increases in modulus due to the interaction between the different blend components correlated to previous findings when using oligo(lactic acid)-g-starch as a compatibilizer in PLA/TPS blends [37], and miscible PLA, TPS, and PCL binary and ternary blends [15]. 

## 4. Conclusions

In this study, TPS was chemically modified in situ with L-LA and PBS chains via two different pathways. The in situ step-by-step reactions of pathway I produced the relatively ordered structure of PLLA-TPS-PBS-I, whereas pathway II created the random structures of PLLA-TPS-PBS-II from the in situ one-pot reactions. The chemical shift correlation in 2D NMR-HMBC mode illustrated the possible obtained structures (**1**), (**2**), and (**3**), which directly manipulated the consequent morphologies and physical properties of the PLA/PLLA-TPS-PBS/PBS ternary blends. The varied feeding molar ratio of the L-LA/starch and the PBS chain length played key roles in multi-phase dispersion and homogeneity improvement. The improved miscibility of the PLA/TPS/PBS ternary blend was successfully achieved by replacing TPS with PLLA-TPS-PBS-I or -II copolymers as the interphase-tuning molecules. The different structures of the obtained PLLA-TPS-PBS copolymers affected crystallization acceleration in the PLA/TPS/PBS ternary blend. PLLA-TPS-PBS-I only promoted PBS crystallization when added, while the random PLLA-TPS-PBS-II copolymers assisted both PLA and PBS crystallization. Finally, PLA/PLLA-TPS-PBS/PBS ternary blend strength and flexibility improved considerably. These results highlight their potential use in fully compostable or food contact packaging. Additionally, this less complicated process for copolymer preparation can be applied in industrial scale production.

## Figures and Tables

**Table 1:** Feeding molar ratios and sample codes of PLLA-TPS-PBS copolymers.

Pathway	Feeding Molar Ratio		PBS with Different MWs	Sample Code
	L-LA	Starch		
I	0.05	1	PBS1	PLLA0.05-TPS-PBS1–I
I	0.05	1	PBS2	PLLA0.05-TPS-PBS2–I
I	0.2	1	PBS1	PLLA0.2-TPS-PBS1–I
I	0.2	1	PBS2	PLLA0.2-TPS-PBS2–I
II	0.05	1	PBS1	PLLA0.05-TPS-PBS1–II
II	0.05	1	PBS2	PLLA0.05-TPS-PBS2–II
II	0.2	1	PBS1	PLLA0.2-TPS-PBS1–II
II	0.2	1	PBS2	PLLA0.2-TPS-PBS2–II

**Table 2:** Thermal properties of PLA/PLLA-TPS-PBS/PBS blends.

Sample	T_g_ (°C)	T_cc,PLA_ (°C)	T_m,PBS_ (°C)	T_m,PLA_ (°C)	X_c,PLA_ (%)	X_c,PBS_ (%)
PLA	53.9	124.5	-	152.2, 156.2	3.70	-
PBS	-	-	115.9	-	-	66.80
PLA/PBS (50/50)	54.8	104.2	114.5	145.2, 152.6	25.03	35.72
PLA/TPS/PBS (35/30/35)	53.9	98.6	112.4	141.1, 147.5	24.66	24.47
PLA/PLLA0.05-TPS-PBS1–I/PBS	50.4	93.6	111.2	139.8, 149.3	16.72	57.08
PLA/PLLA0.05-TPS-PBS2–I/PBS	51.9	92.5	103.9, 111.3	138.7, 148.7	19.55	61.58
PLA/PLLA0.2-TPS-PBS1–I/PBS	50.4	89.7, 94.8	104.2, 111.2	137.9, 148.8	22.60	61.65
PLA/PLLA0.2-TPS-PBS2–I/PBS	48.6	89.5, 92.3	103.8, 111.3	138.7, 148.5	20.70	63.26
PLA/PLLA0.05-TPS-PBS1–II/PBS	52.5	92.0	110.1	138.4, 147.7	28.94	63.40
PLA/PLLA0.05-TPS-PBS2–II/PBS	53.5	96.9	113.0	142.3, 150.1	33.25	53.52
PLA/PLLA0.2-TPS-PBS1–II/PBS	44.2	81.8, 89.1	109.1	135.0, 144.8	30.18	63.34
PLA/PLLA0.2-TPS-PBS2–II/PBS	49.7	93.0	112.0	138.4, 149.1	23.92	62.22

## Data Availability

Not applicable.

## Supplementary Materials

The following supporting information can be downloaded at: https://www.mdpi.com/article/10.3390/polym14040825/s1, Figure S1: ^1^H- and ^13^C-NMR spectra of PLLA0.2-TPS-PBS2–I and PLLA0.2-TPS-PBS2–II copolymers in CDCl_3_ solvent, Figure S2: DSC thermograms of (a) PLA/PBS 50/50, and (b) PLA/PLLA0.05-TPS-PBS1–II/PBS, (c) PLA/PLLA0.05-TPS-PBS2–II/PBS, (d) PLA/PLLA0.2-TPS-PBS1–II/PBS, and (e) PLA/PLLA0.2-TPS-PBS2–II/PBS ternary blend at weight ratio of 35/30/35.
