# Supplementary material for: In Situ Chemical Modification of Thermoplastic Starch with Poly(L-lactide) and Poly(butylene succinate) for an Effectively Miscible Ternary Blend
Source: Polymers (Basel). 2022 Feb 21;14(4):825. doi: 10.3390/polym14040825 (PMC8880544; doi:10.3390/polym14040825)
Supplement: Supplementary file 1 [file polymers-14-00825-s001.zip › polymers-1553997-supplementary.pdf]

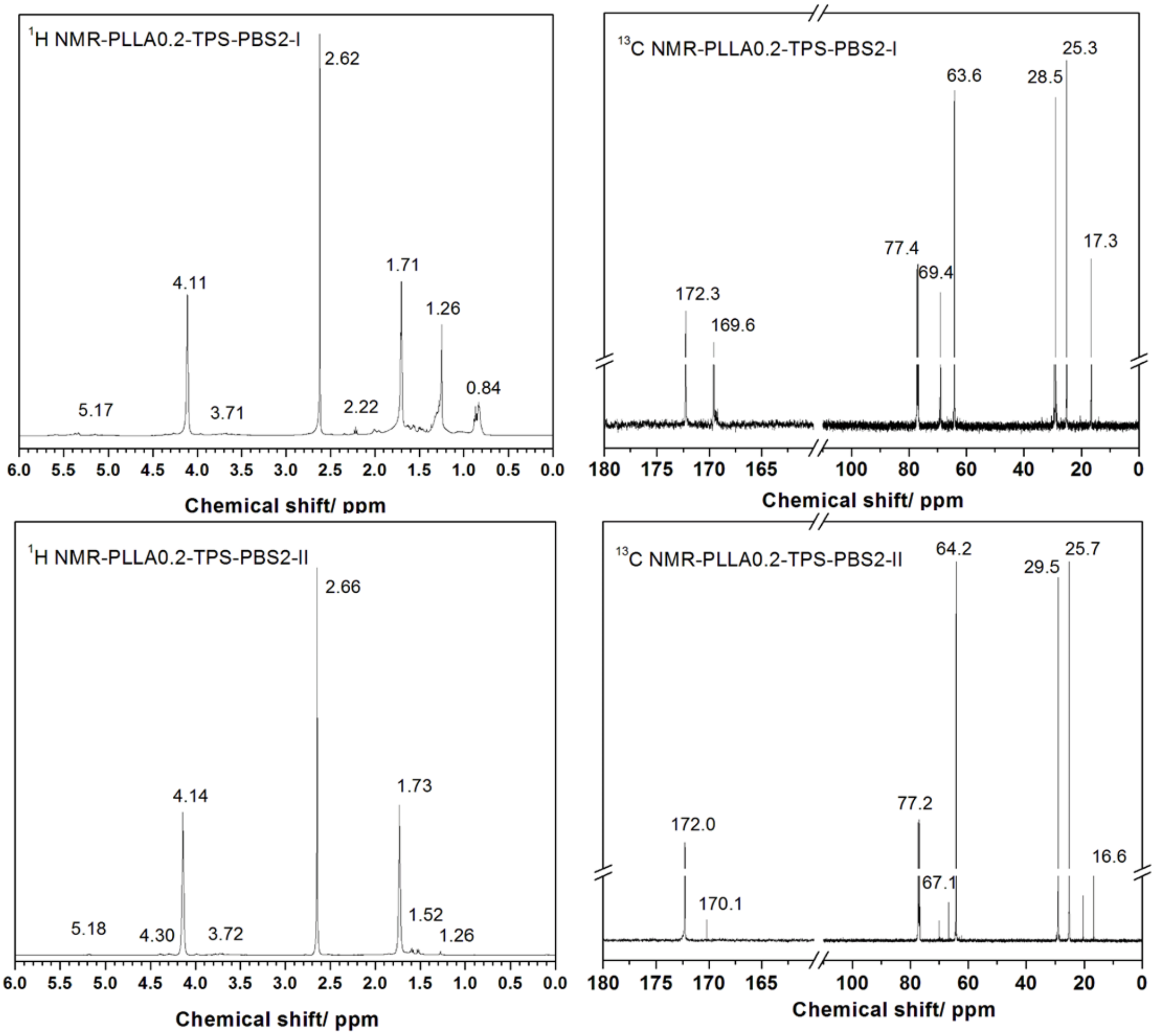

Figure S1. ^{1}H- and ^{13}C-NMR spectra of PLLA0.2-TPS-PBS2–I and PLLA0.2-TPS-PBS2–II copolymers in CDCl3 solvent.

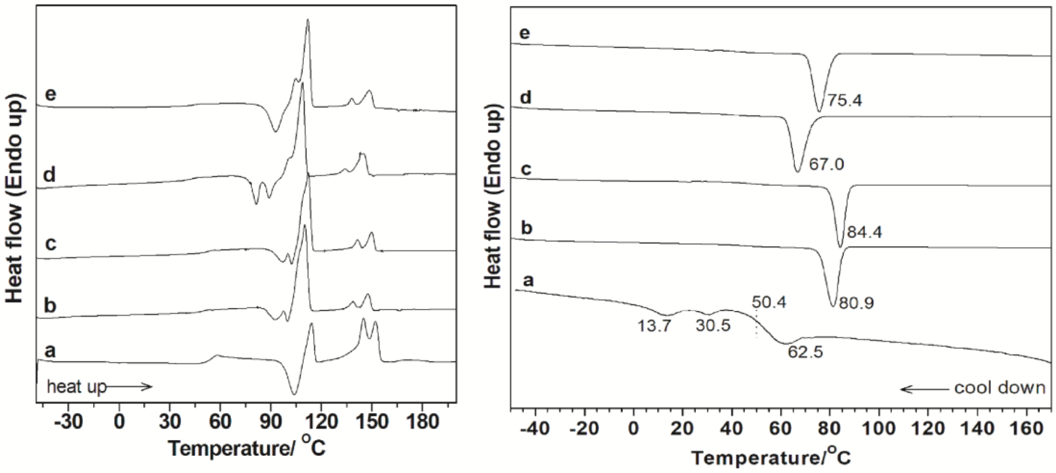

Figure S2. DSC thermograms of (A) 2nd heating and (B) cooling scans: (a) PLA/PBS 50/50, and (b) PLA/PLLA**0.05**-TPS-PBS**1**–**II**/PBS, (c) PLA/PLLA**0.05**-TPS-PBS**2**–**II**/PBS, (d) PLA/PLLA**0.2**-TPS-PBS**1**–**II**/PBS, and (e) PLA/PLLA**0.2**-TPS-PBS**2**–**II**/PBS ternary blend at weight ratio of 35/30/35.
